# Meta-Analysis for the Association between Polymorphisms in Interleukin-17A and Risk of Coronary Artery Disease

**DOI:** 10.3390/ijerph13070660

**Published:** 2016-06-30

**Authors:** Mei-Hua Bao, Huai-Qing Luo, Ju Xiang, Liang Tang, Li-Ping Dong, Guang-Yi Li, Jie Zeng, Jian-Ming Li

**Affiliations:** 1 Department of Anatomy, Histology and Embryology, Institute of Neuroscience, Changsha Medical University, Changsha 410219, China; luohuaiqing@163.com (H.-Q.L.); Xiang.ju@foxmail.com (J.X.); tlcool318@163.com (L.T.); ddongliping@163.com (L.-P.D.); liguangyi1977@163.com (G.-Y.L.); zengjie84117@163.com (J.Z.); 2 Department of Neurology, Xiangya Hospital, Central South University, Changsha 410008, China

**Keywords:** IL-17A, rs3748067, rs2275913, rs8193037, coronary artery disease, polymorphism, meta-analysis

## Abstract

Coronary artery disease (CAD) is a disease which has become a leading cause of death worldwide. The polymorphisms in Interleukin-17 (IL-17A), including rs2275913, rs3819024, rs3819025, rs3748067, rs8193037, rs4711998, and rs8193036, have been found to be probably associated with the risk of CAD. However, the results were inconsistent and inconclusive. The present study performed a meta-analysis to get a more precise and comprehensive estimation of the association between the IL-17A polymorphisms and CAD risk. The Pubmed, Embase, Cochrane Central Register of Controlled Trials, Chinese National Knowledge Infrastructure, and Chinese Biomedical Literature Databases were searched for related studies. A total of six studies, including 3542 cases and 3212 controls, were identified for the meta-analysis. The main findings of the present meta-analysis show that the TT genotype of IL-17A rs3748067 is associated with a significant lower risk of CAD in the homozygous model odds ratio (OR) (OR = 0.37) in Asians. No significant association was found for rs2275913, rs3819024, rs3819025, rs8193037, rs4711998, and rs8193036 with CAD susceptibility in the overall analysis. However, subgroup analysis indicated a significant decreased risk of CAD for the GG genotype and G allele of rs2275913 in a small sample size group, and a higher risk of CAD for the GG genotype and G allele of rs8193037 in a heterozygous model (OR = 1.56), dominant model (OR = 1.54), and allelic model (OR = 1.47) in Asians. In conclusion, the current meta-analysis suggests a significant relationship between rs3748067, rs8193037, and CAD in Asians, while for rs2275913, rs3819024, rs3819025, rs4711998, rs8193036, no such relations were found. Thus, IL-17A rs3748067 and rs8193037 might be recommended as a predictor for susceptibility of CAD for Asians. However, the results of this meta-analysis are hypothesis-generating results which should be interpreted with caution because of the heterogeneity and publication bias among study designs.

## 1. Introduction

It is well known that atherosclerosis is a chronic inflammatory disease, which has become the major cause of cardiovascular diseases (CVDs), including coronary artery disease (CAD), ischemic stroke, cerebral stroke, and peripheral vascular disease [1]. Among these CVDs, CAD is a disease which represents a leading cause of death worldwide. According to the Chinese Cardiovascular Disease Epidemiology Report 2015, the annual CAD mortality rate was 105.7 per 100,000 worldwide and 70.1 per 100,000 in China. As a principal clinical manifestation of atherosclerosis, the risk factors of CAD include age, smoking, diabetes mellitus, and hyperlipidemia, etc. The genetic mutant was also found to play important roles in CAD susceptibility.

Interleukin-17 (IL-17) is a novel family cytokine that consists of six protein members (from IL-17A to IL-17F), which plays an important role in many chronic inflammatory diseases [2,3]. It has been demonstrated that atherosclerotic plaque shows higher expression of IL-17A [4]. After myocardial ischemia/reperfusion injuries, the expression of IL-17A and IL-17F also increase [5]. Recent studies indicated the IL-17A polymorphisms be connected with the risk of developing atherosclerosis and CAD [6,7,8,9,10]. Several polymorphisms in IL-17A, such as rs2275913, rs3819024, rs3819025, rs3748067, rs8193037, rs4711998, and rs8193036 have been found to be related with the risk of CAD [6,7,8,9,10]. According to recent data, the AA and GA + AA genotype of rs2275913, CC, and TC + CC of rs3748067 are thought to be probably associated with the risk of CAD by some research [7,8]. However, the results were inconsistent and inconclusive [9,10,11]. Thus, in the present study, we included a total of six studies, with 3542 cases and 3212 controls, to get a more precise and comprehensive estimation of the association between these polymorphisms and CAD.

## 2. Experimental Section

### 2.1. Publication Search Strategy and Inclusion Criteria

We systematically searched the published studies in the following electronic databases: Pubmed, Embase, Cochrane Central Register of Controlled Trials (CENTRAL), Chinese National Knowledge Infrastructure (CNKI), and Chinese Biomedical Literature Database (CBM). The searched terms were (“Coronary artery disease”, or “CAD”), (“interleukin-17A” or “IL-17a”), and (“polymorphism” or “mutation” or “SNP” or “single nucleotide polymorphism”), without restrictions on language. The deadline for publication was 30 April 2016. All of the results from the databases were screened. Firstly, we screened the title. If the titles fulfilled our criteria, we then screened the abstract. We retrieved the full text if the abstract matched our interest. The references of all eligible studies were retrieved manually for other potentially relevant studies. We contacted the authors for related data that were unavailable in the original publications.

Inclusion criteria: (a) case-control design; (b) the association of interleukin-17A polymorphisms and CAD risks should be evaluated; (c) the data in the publication were sufficient for the present estimation. Studies were excluded if any of the following applies: (1) repeat publications, abstracts, letters or reviews; (2) studies not meeting all of the inclusion criteria.

### 2.2. Data Extraction

We extracted information from each eligible publication manually by two investigators independently. For each study, the extracted information included: first authors’ name, publishing year, country, ethnicity, genotyping method, the source of controls, sex and age match, and genotype numbers of cases and controls. If we met discrepancies during the data extraction, they would be resolved by a consensus achieved by the third author.

### 2.3. Quality Assessment

The quality of the included studies was evaluated according to the predefined scale for quality assessment [12]. The score scale includes the following aspects: source of cases, source of controls, specimens used for determining genotypes, total sample size, and evidence of Hardy-Weinberg equilibrium (HWE). The quality scores range from 0–15. Reports scoring <10 were classified as “low quality”, and those ≥10 as “high quality”. The quality evaluation was performed by two authors independently. Consensus was held to resolve any discrepancies in the assessment process.

### 2.4. Statistical Methods

*χ*^2^-test was used to evaluate the HWE of the control group polymorphism. If *p* < 0.05, it was considered to be deviated from HWE. To evaluate the association between IL-17A polymorphisms and CAD risk, the crude odds ratio (OR) with 95% confidence interval (CI) was used. The pooled ORs were calculated using homozygous, heterozygous, dominant, and allelic genetic model. The statistical significance was determined by the *Z*-test, and the *p*-value was adjusted using Bonferroni’s correction. *p* < 0.05 was considered to be statistically significant. Subgroup analysis was conducted by the sample size and ethnicity. Total samples minor than 1000 will be treated as small, and large, otherwise.

The statistical heterogeneity between studies was evaluated by an *I*-square statistical test, which was not dependent on the number of studies in the meta-analysis [13]. If there was an obvious heterogeneity among the studies (*I*^2^ > 50%), the random-effects model (the DerSimonian and Laird method) was used for the meta-analysis [14]. Otherwise, the fixed-effect model using the Mantel-Haenszel method was adopted [15]. Sensitivity analysis was performed to assess the effect of individual study on pooled results and the stability of results. The publication bias was detected with Begg’s funnel plot and Egger’s linear regression method, and *p* < 0.05 was considered to be statistically significant [16]. All statistical analyses were performed using the STATA 12.0 software (StataCorp, College Station, TX, USA) and Revman 5.3 (The Cochrane Collaboration, Oxford, UK).

## 3. Results and Discussion

### 3.1. Characteristics of Eligible Studies

**Figure 1:**
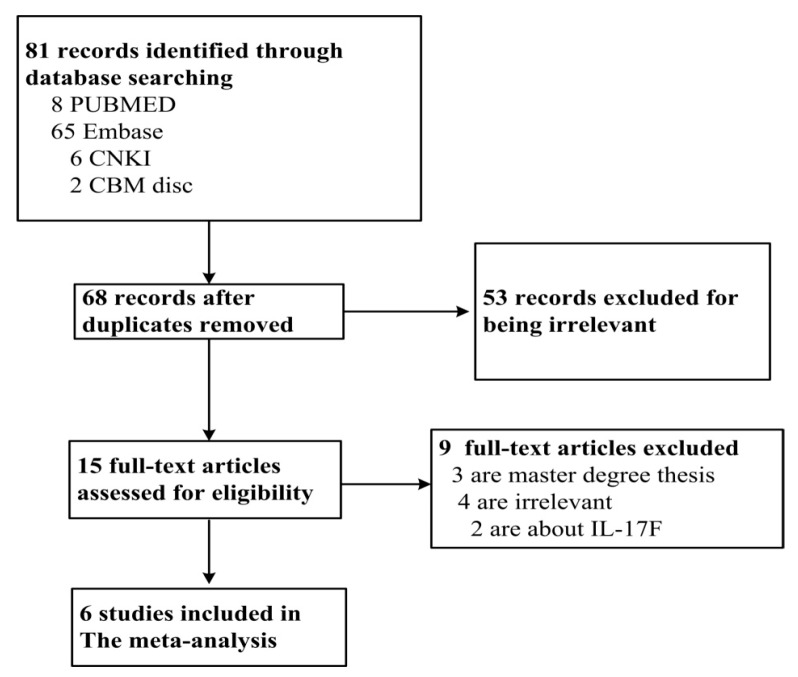
PRISMA flowchart of study inclusion and exclusion.

A total of 68 studies were obtained from the literature search after duplicates were removed. Among them, 57 studies were excluded for irrelevance, three were master’s degree theses, two for predicting IL-17A polymorphisms and ischemic stroke. Finally, six studies met the criteria, including 3542 cases and 3212 controls [7,8,9,10,11,17]. Among them, five studies described the associations between IL-17A rs2275913 and CAD, and three studies described the associations between IL-17A rs8193037 and CAD, two studies for each described association between rs3819025, rs3748067, rs3819024 and CAD, and one study for each described association between IL-17A rs4711998, rs8193036, and CAD. The PRISMA flowchart is shown in Figure 1 and the information for included studies is presented in Table 1.

### 3.2. Results of Meta-Analysis

**Figure 2:**
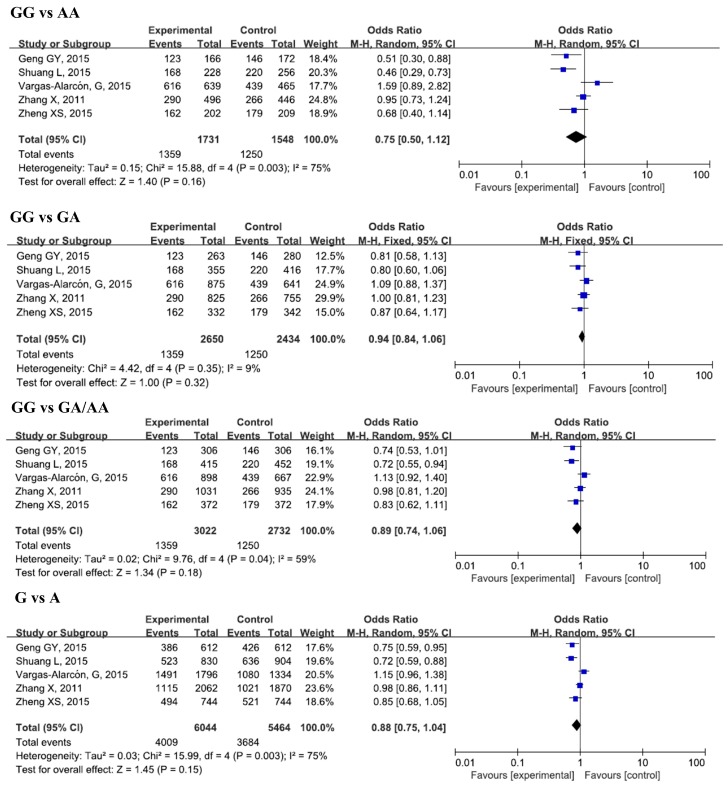
Forest plots of odds ratios for the association of IL-17A rs2275913 with risk of CAD.

**Figure 3:**
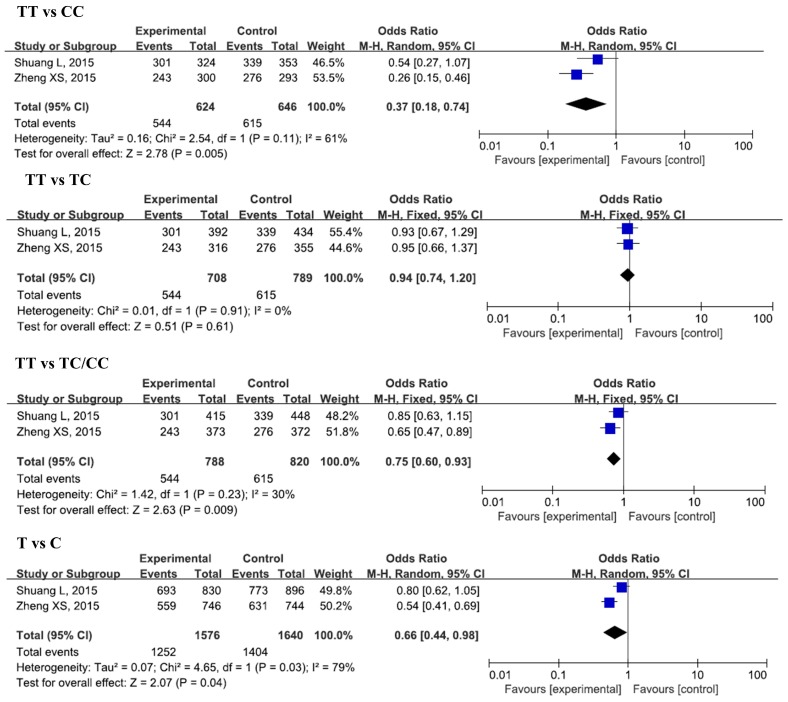
Forest plots of odds ratios for the association of IL-17A rs3748067 with risk of CAD.

**Figure 4:**
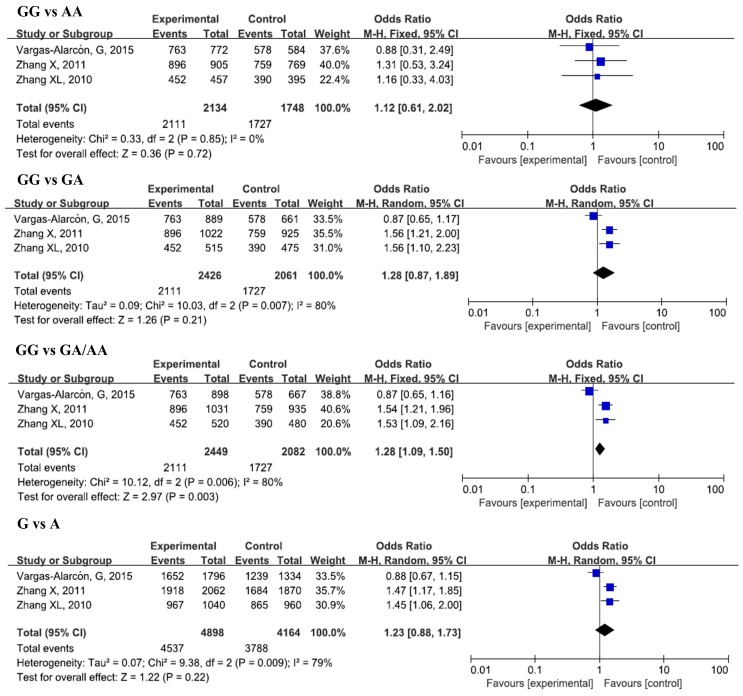
Forest plots of odds ratios for the association of IL-17A rs8193037 with risk of CAD.

The results of the meta-analysis for the associations between IL-17A rs2275913, rs3819024, rs3819025, rs8193037, rs3748067, rs4711998, rs8193036, and CAD risks were shown in Table 2, Figure 2, Figure 3 and Figure 4, and Supplementary Figures S1 and S2.

#### 3.2.1. IL-17A rs2275913 and CAD

Five studies, with 3022 cases and 2732 controls, were included in this analysis. No significant associations were found between rs2275913 and CAD susceptibility in the overall analysis for all genetic models. However, when we conducted a subgroup analysis by sample size, significant decreases in risk of CAD for the GG genotype and G allele in a small sample size group in all genetic models was found (GG vs. AA, OR = 0.53, 95% CI = 0.40–0.71, *p_adj_* < 0.0001; GG vs. GA/AA, OR = 0.76, 95% CI = 0.64–0.90, *p_adj_* = 0.007; G vs. A, OR = 0.77, 95% CI = 0.68–0.87, *p_adj_* < 0.0001). In the subgroup analysis by ethnicity, we did not find any remarkable differences between Asians and Caucasians after Bonferroni’s adjustment of *p*-value.

#### 3.2.2. IL-17A rs8193037 and CAD

Three studies, with 2449 cases and 2082 controls, were included in this analysis. No significant associations were found in the overall analysis for all genetic models. In the subgroup analysis by ethnicity, a significant increase in CAD susceptibility was found for the GG genotype and G allele in a heterozygous model (GG vs. GA, OR = 1.56, 95% CI = 1.27–1.91, *p_adj_* < 0.0001), dominant model (GG vs. GA/AA, OR = 1.54, 95% CI = 1.26–1.88, *p_adj_* < 0.0001), and allelic model (G vs. A, OR = 1.47, 95% CI = 1.22–1.76, *p_adj_* < 0.0001) in Asians.

#### 3.2.3. IL-17A rs3748067 and CAD

Two studies, with 788 cases and 820 controls, were included in this analysis. A significant decrease in CAD risk was found in the homozygous model (TT vs. CC, OR = 0.37, 95% CI = 0.18–0.74, *p_adj_* = 0.04). No significant associations were found between these SNPs and CAD risk in other genetic models.

#### 3.2.4. IL-17A rs3819024, rs3819025 and CAD

For the association between rs3819024, rs3819025, and CAD, two studies were included for each of them (1929 cases and 1602 controls for rs3819024, and 1446 cases and 1383 controls for rs3819025). No significant relationship was found between these SNPs and CAD risk in all genetic models (Figures S1 and S2).

#### 3.2.5. IL-17A rs4711998, rs8193036, and CAD

One study with 1031 cases and 935 controls was included in the analysis for rs4711998 and CAD; other study with 895 cases and 664 controls, was included in the analysis for rs8193036. No significant associations were found between these SNPs and CAD susceptibility in the overall analysis.

### 3.3. Sources of Heterogeneity

Since a significant heterogeneity was found for IL-17A rs2275913, we performed a subgroup analysis by sample size and ethnicity to explore the source of heterogeneity. The results indicate that the sample size was the source of heterogeneity for rs2275913 in all genetic models. Ethnicity also took the responsibility in heterogeneity and dominant models. For IL-17A rs8193037, ethnicity is the source of the heterogeneity in all genetic models.

### 3.4. Sensitivity Analysis

The influence of each study on the pooled ORs and 95% CIs was evaluated by excluding one single study at a time using STATA 12.0 software. No significant altered pooled ORs were found in all genetic models for IL-17A rs2275913 (data not shown).

### 3.5. Publication Bias

**Figure 5:**
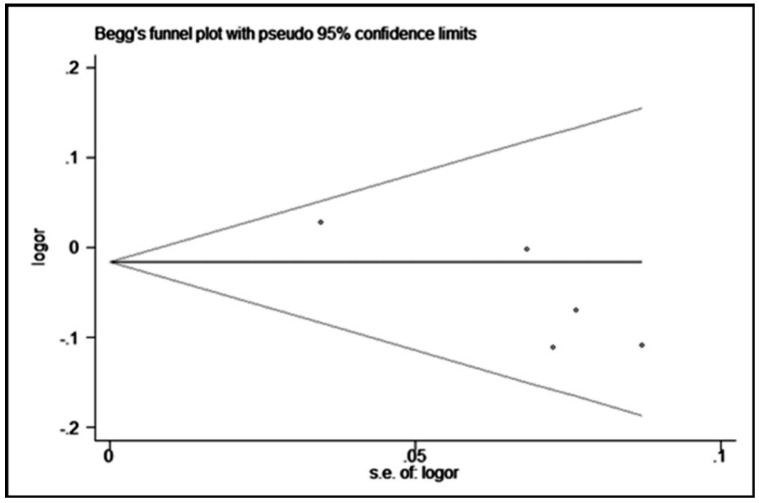
Begg’s funnel plot with pseudo 95% confidence limits for a heterozygous model (GG vs. GA) of rs2275913.

We performed the Begg’s funnel plot and Egger’s test to evaluate the publication bias. The *p* values for Begg’s and Egger’s tests are shown in Table 3. Obvious publication bias was observed for rs2275913 in heterozygous, dominant, and allelic models in Egger’s test. These results were also demonstrated by the shape of the funnel plot (only the heterozygous model results are shown, in Figure 5).

## 4. Discussion

The main findings of the present meta-analysis show that the TT genotype of IL-17A rs3748067 was associated with a significant lower risk of CAD in a homozygous model (OR = 0.37) in Asians. No significant association was found for rs2275913, rs3819024, rs3819025, rs8193037, rs4711998, and rs8193036 with CAD susceptibility in overall analysis. However, a subgroup analysis indicated a significantly decreased risk of CAD for the GG genotype and G allele of rs2275913 in small sample size groups. A higher risk of CAD for the GG genotype and G allele of rs8193037 was observed in a heterozygous model (OR = 1.56), dominant model (OR = 1.54), and allelic model (OR = 1.47) in Asians.

Previous studies have reported the importance of inflammation in the risk of atherosclerosis [18]. IL-17 is produced by activated CD4+ T cells (Th17 cells) and other leukocytes. IL-17 family contains six members (IL-17A~F) [19]. IL-17A is one of the most important members, which induces the production of multiple cytokines, chemokines, and adhesion molecules in atherogenic cells. As a result, IL-17A was demonstrated to govern mononuclear cell accumulation and cell death, mobilization, recruitment, and activation of macrophages in atherosclerotic lesions, which eventually cause atherogenesis, atherosclerotic plaque disruption, and thrombosis [20,21]. Several studies indicated the correlation between IL-17A and CRP in different diseases, such as cardiovascular disease [22], inflammatory bowel disease [23], Cystic Echinococcosis [24], Behcet’s disease [25], etc. It was also reported that IL-17A triggered the inflammatory mediator Interleukin-1α (IL-1α) through NLR family pyrin domain containing 3 (NLRP3) inflammasome and NF-κB signal [26]. In ischemia/reperfusion injury in cardiac myocytes, enhanced IL-17A increased the pro-inflammatory targets, such as chemokine (C-X-C motif) ligand 1 and interleukin-6, through mitogen-activated protein kinases. Inhibition of the IL-17 pathway reduced necrosis and apoptosis in myocytes [5]. However, there are no studies investigating the relationship between these SNP and inflammation. Therefore, whether these SNPs in IL-17A have effects on these pro-inflammatory factors, as well as the pathways, still needs further investigation.

Recently, several SNPs in IL-17A have been studied to investigate their relations with CAD risk. rs2275913 has gained the greatest attention in these involved studies, some of which found a significant association between rs2275913 and CAD [7,8], while others denied it [9,10,11]. Our meta-analysis did not find any relations between rs2275913 and CAD in overall analysis, but lower risk of CAD was found in GG genotype in small sample size groups when subgroup analysis was conducted. Since the small sample studies sometimes have small-study effects [27], our results on the relationship between rs2275913 and CAD should be considered with caution.

In Asians, we also observed a higher risk of CAD for the GG genotype and G allele of rs8193037. However, only one study was included for the evaluation in Caucasians. Further studies are still needed to confirm the associations in other ethnicities. We also found a significant association between rs3748067 and CAD, but significant deviation from HWE (*p* < 0.05) was found for both of the two included studies. Furthermore, the two studies were all conducted among Asians. Therefore, further studies with larger sample sizes are required to confirm the results.

Significant heterogeneity was found in the meta-analysis for rs2275913, rs8193037, rs3748067, rs3819025, and rs3819024. Thus, we conducted subgroup analysis by sample size and ethnicity and found that both of them were the sources of the heterogeneity for rs2275913, and ethnicity was the source of the heterogeneity for rs8193037. However, only one study was included in the Caucasian group, and two studies were included in a large sample size group of rs2275913. And for rs8193037, a total of three studies were included, with two in Asians and one in Caucasians. Further studies are needed to confirm these results. In the sensitivity analysis, no significant changes were found after omitting each study at a time, indicating the relative stability and credibility of the results of our meta-analysis.

We found an obvious asymmetry in funnel plots, and a significant *p*-value for rs2275913 through Egger’s test in the present study. According to the Cochrane Handbook (version 5.1.0, Section 10), the cause for the asymmetry in funnel plots includes selection bias (publication bias, selective outcome reporting), small-sample effects, true heterogeneity, artifacts, and chance. In the present meta-analysis, most of the included studies were of small size. After a comprehensive literature search, all published studies were included in the present study. No large consortia or GWAS data are available yet. Therefore, the small-sample effects might be one reason for this bias. Furthermore, we have assessed the quality of all included studies, and they are all of high quality. However, only studies in English or Chinese have been searched and included. There might be studies in other languages which are not included in the present analysis. Based on the Cochrane Handbook, tests for funnel plot asymmetry should be used only when there are at least 10 studies included, because when there are fewer studies the power of the test is too low to distinguish chance from real asymmetry. In the present test, only five studies are included, which might be another reason for the asymmetry.

We included six studies in our present meta-analysis, while only one study was conducted in Caucasians, which estimated the associations between rs2275913, rs8193037, and rs3819024 with risk of CAD. However, no obvious relationships were found in all of these SNPs. The positive results in our meta-analysis are all related with Asians. Thus, the conclusion drawn in the present meta-analysis might be only of generalizability for Asians.

The results of the present meta-analysis should be interpreted carefully because of the following limitations. Firstly, the number of patients was relatively small, and may influence the outcomes. After a very comprehensive literature search from several different databases, only six studies including 3542 cases and 3212 controls were included in the present meta-analysis, and for rs8193037, rs3748067, rs3819025, and rs3819024, even fewer studies, each less than three were included. Secondly, the clinicopathological characteristics or disease subtypes are limited in most of these studies. Thirdly, the ethnicity of the subjects is predominantly Asiatic. Only one study, with 898 cases and 667 controls, was committed in Caucasians, and the last limitation is that CAD is a multi-factorial disease influenced by both genetic and environmental factors. The gene-gene and gene-environment interactions may play important roles in the functions of these polymorphisms, but most studies lack the information on environmental exposure and multiple SNPs in haplotypes.

## 5. Conclusions

In conclusion, the current meta-analysis suggests a lower risk of CAD for the TT genotype of IL-17A rs3748067 in Asians, and a higher risk of CAD for the GG genotype and G allele of rs8193037 in Asians. A decreased risk of CAD for the GG genotype and G allele of rs2275913 were observed in a small sample size group, while rs3819024, rs3819025, and rs4711998 are not associated with the risk of CAD. Thus, IL-17A rs3748067 and rs8193037 might be recommended as predictors for susceptibility of CAD in Asians. However, the results of this meta-analysis are hypothesis-generating results which should be interpreted with caution because of the heterogeneity and publication bias among study designs. Further study is needed to evaluate the association between these SNPs and CAD, especially in a large sample size, in Caucasians, and with clinicopathological characteristics.

## Figures and Tables

**Table 1:** Characteristics of eligible studies included in the meta-analysis.

IL-17A rs2275913 and CAD												
Author	Year	Country	Ethnicity	Genotyping Methods	Sex Ratio (Male:Female) (Case/Control)	Age (Case/Control)	Quality Score	Sample Size (Case/Control)	GG	GA	AA	HWE of Control
									(Case/Control)	(Case/Control)	(Case/Control)	
Shuang, L. [8]	2015	China	Asian	PCR-RFLP	321:94/265:183	59.20 ± 10.85/ 60.10 ± 10.35	13	415/452	168/220	187/196	60/36	0.401
Zheng, X.S. [11]	2015	China	Asian	PCR-RFLP	280:92/280:92	62.15 ± 11.30/ 61.74 ± 10.95	13	372/372	162/179	170/163	40/30	0.398
Geng, G.Y. [7]	2015	China	Asian	PCR-RFLP	234:72/234:72	61.5 ± 10.5/ 60.3 ± 9.8	13	306/306	123/146	140/134	43/26	0.541
Vargas-Alarcón, G. [9]	2015	Mexico	Caucasian	Taqman	253:414/744:156	54 [49,58]/ 52 [47,58]	14	898/667	616/439	259/202	23/26	0.648
Zhang, X. [10]	2011	China	Asian	Sequencing PCR	612:419/524:411	55.9 ± 10.1/ 55.2 ± 10.5	12	1031/935	290/266	535/489	206/180	0.093
**IL-17A rs3819025 and CAD**												
**Author**	**Year**	**Country**	**Ethnicity**	**Genotyping Methods**	**Sex Ratio (Male:Female) (Case/Control)**	**Age (Case/Control)**	**Quality Score**	**Sample Size (Case/Control)**	**AA**	**AG**	**GG**	**HWE of Control**
									**(Case/Control)**	**(Case/Control)**	**(Case/Control)**	
Shuang, L. [8]	2015	China	Asian	PCR-RFLP	321:94/265:183	59.20 ± 10.85/ 60.10 ± 10.35	13	415/448	179/212	177/180	59/56	0.07
Zhang, X. [10]	2011	China	Asian	Sequencing PCR	612:419/524:411	55.9 ± 10.1/ 55.2 ± 10.5	12	1031/935	635/554	358/342	38/39	0.125
**IL-17A rs3748067 and CAD**												
**Author**	**Year**	**Country**	**Ethnicity**	**Genotyping Methods**	**Sex Ratio (Male:Female) (Case/Control)**	**Age (Case/Control)**	**Quality Score**	**Sample Size (Case/Control)**	**TT**	**TC**	**CC**	**HWE of Control**
									**(Case/Control)**	**(Case/Control)**	**(Case/Control)**	
Shuang, L. [8]	2015	China	Asian	PCR-RFLP	321:94/265:183	59.20 ± 10.85/ 0.10 ± 10.35	10	415/448	301/339	91/95	23/14	0.03
Zheng, X.S. [11]	2015	China	Asian	PCR-RFLP	280:92/280:92	62.15 ± 11.30/ 61.74 ± 10.95	10	372/372	243/276	73/79	57/17	<0.0001
**IL-17A rs3819024 and CAD**												
**Author**	**Year**	**Country**	**Ethnicity**	**Genotyping Methods**	**Sex Ratio (Male:Female) (Case/Control)**	**Age (Case/Control)**	**Quality Score**	**Sample Size (Case/Control)**	**AA**	**AG**	**GG**	**HWE of Control**
									**(Case/Control)**	**(Case/Control)**	**(Case/Control)**	
Vargas-Alarcón, G. [9]	2015	Mexico	Caucasian	Taqman	253:414/744:156	54 [49,58]/ 52 [47,58]	14	898/667	615/434	259/209	24/24	0.851
Zhang, X. [10]	2011	China	Asian	Sequencing PCR	612:419/524:411	55.9 ± 10.1/ 55.2 ± 10.5	12	1031/935	230/219	542/470	259/246	0.850
**IL-17A rs8193037 and CAD**												
**Author**	**Year**	**Country**	**Ethnicity**	**Genotyping Methods**	**Sex Ratio (Male:Female) (Case/Control)**	**Age (Case/Control)**	**Quality Score**	**Sample Size (Case/Control)**	**GG**	**GA**	**AA**	**HWE of Control**
									**(Case/Control)**	**(Case/Control)**	**(Case/Control)**	
Vargas-Alarcón, G. [9]	2015	Mexico	Caucasian	Taqman	253:414/744:156	54 [49,58]/ 52 [47,58]	14	898/667	763/578	126/83	9/6	0.125
Zhang, X. [10]	2011	China	Asian	Sequencing PCR	612:419/524:411	55.9 ± 10.1/ 55.2 ± 10.5	12	1031/935	896/759	126/166	9/10	0.784
Zhang XL [17]	2010	China	Asian	PCR-RFLP	291:229/283:197	55.9 ± 10.1/ 55.2 ± 10.5	11	520/480	452/390	63/85	5/5	0.878
**IL-17A rs819336 and CAD**												
**Author**	**Year**	**Country**	**Ethnicity**	**Genotyping Methods**	**Sex Ratio (Male:Female) (Case/Control)**	**Age (Case/Control)**		**Sample Size (Case/Control)**	**TT**	**TC**	**CC**	**HWE of Control**
									**(Case/Control)**	**(Case/Control)**	**(Case/Control)**	
Vargas-Alarcón, G. [9]	2015	Mexico	Caucasian	Taqman	253:414/744:156	54 [49,58]/ 52 [47,58]		898/667	546/409	303/221	46/34	0.561
**IL-17A rs4711998 and CAD**												
**Author**	**Year**	**Country**	**Ethnicity**	**Genotyping Methods**	**Sex Ratio (Male:Female) (Case/Control)**	**Age (Case/Control)**	**Quality Score**	**Sample Size (Case/Control)**	**AA**	**AG**	**GG**	**HWE of Control**
									**(Case/Control)**	**(Case/Control)**	**(Case/Control)**	
Zhang, X. [10]	2011	China	Asian	Sequencing PCR	612:419/524:411	55.9 ± 10.1/ 55.2 ± 10.5	12	1031/935	626/593	349/295	56/47	0.194

PCR-RFLP: Polymerase chain reaction- restriction fragment length polymorphism.

**Table 2:** Pooled ORs and 95% CIs of the association between IL-17A rs2275913, rs3819024, rs3819025, rs8193037, rs3748067, rs4711998, rs8193036, and CAD risks.

IL-17A rs2275913 and CAD													
Genetic Model		N	OR (95% CI)	*p*	*p*_adj_	*I*^2^ (%)	Genetic Model		N	OR (95% CI)	*p*	*p*_adj_	*I*^2^ (%)
GG vs. AA	Overall	5	0.75 (0.50, 1.12)	0.16	-	75	GG vs. GA	Overall	5	0.94 (0.84, 1.06)	0.32	-	9
	Small size	3	0.53 (0.40, 0.71)	<0.0001	<0.0001	0		Small size	3	0.82 (0.69, 0.98)	0.03	0.21	0
	Large size	2	1.15 (0.71, 1.86)	0.57	-	60		Large size	2	1.04 (0.90, 1.21)	0.60	-	0
	Asian	4	0.68 (0.40, 1.14)	0.03	0.21	69		Asian	4	0.89 (0.78, 1.02)	0.10	-	0
	Caucasian	1	1.59 (0.89, 2.82)	0.12	-	N.A		Caucasian	1	1.09 (0.88, 1.37)	0.42	-	N.A
GG vs. GA/AA	Overall	5	0.89 (0.74, 1.06)	0.18	-	59	G vs. A	Overall	5	0.88 (0.75, 1.04)	0.15	-	75
	Small size	3	0.76 (0.64, 0.90)	0.001	0.007	0		Small size	3	0.77 (0.68, 0.87)	<0.0001	<0.0001	0
	Large size	2	1.05 (0.91, 1.21)	0.50	-	0		Large size	2	1.05 (0.90, 1.22)	0.57	-	50
	Asian	4	0.83 (0.71, 0.98)	0.02	0.14	33		Asian	4	0.83 (0.71, 0.97)	0.02	0.14	65
	Caucasian	1	1.13 (0.92, 1.40)	0.25	-	N.A		Caucasian	1	1.15 (0.96, 1.38)	0.14	-	N.A
**IL-17A rs8193037 and CAD**													
**Genetic Model**		**N**	**OR (95% CI)**	***p***	***p*_adj_**	***I*^2^ (%)**	**Genetic Model**		**N**	**OR (95% CI)**	***p***	***p*_adj_**	***I*^2^ (%)**
GG vs. AA	Overall	3	1.12 (0.61, 2.02)	0.72	-	0	GG vs. GA	Overall	3	1.28 (0.87, 1.89)	0.21	-	80
	Asian	2	1.26 (0.60, 2.61)	0.54	-	0		Asian	2	1.56 (1.27, 1.91)	<0.0001	<0.0001	0
	Caucasian	1	0.88 (0.31, 2.49)	0.81	-	N.A		Caucasian	1	0.87 (0.65, 1.17)	0.36	-	N.A
GG vs. GA/AA	Overall	3	1.27 (0.87, 1.85)	0.21	-	80	G vs. A	Overall	3	1.23 (0.88, 1.73)	0.22	-	79
	Asian	2	1.54 (1.26, 1.88)	<0.0001	<0.0001	0		Asian	2	1.47 (1.22, 1.76)	<0.0001	<0.0001	0
	Caucasian	1	0.87 (0.65, 1.16)	0.35	-	N.A		Caucasian	1	0.88 (0.67, 1.15)	0.35	-	N.A
		**IL-17A rs3819024 and CAD**							**IL-17A rs3819025 and CAD**						
**Genetic Model**		**N**	**OR (95% CI)**	***p***	***p*_adj_**	***I*^2^ (%)**			**N**	**OR (95% CI)**	***p***	***p*_adj_**	***I*^2^ (%)**
AA vs. GG		2	1.06 (0.84, 1.33)	0.65	-	16		AA vs. GG	2	0.95 (0.70, 1.30)	0.76	-	32
AA vs. GA		2	1.02 (0.82, 1.28)	0.85	-	51		AA vs. GA	2	1.02 (0.87, 1.19)	0.82	-	48
AA vs. GA/GG		2	1.05 (0.85, 1.29)	0.68	-	51		AA vs. GA/GG	2	0.98 (0.76, 1.27)	0.90	-	62
A vs. G		2	1.05 (0.95, 1.16)	0.37	-	39		A vs. G	2	0.99 (0.80, 1.22)	0.89	-	65
		**IL-17A rs8193036 and CAD**							**IL-17A rs3748067 and CAD**						
**Genetic Model**		**N**	**OR (95% CI)**	**P**	***p*_adj_**	***I^2^* (%)**		**Genetic Model**	**N**	**OR (95% CI)**	**P**	***p*_adj_**	***I^2^* (%)**
TT vs. CC		1	0.99 (0.62, 1.57)	0.95	-	N.A		TT vs. CC	2	0.37 (0.18, 0.74)	0.005	0.04	61
TT vs. TC		1	0.97 (0.78, 1.21)	0.81	-	N.A		TT vs. TC	2	0.94 (0.74, 1.20)	0.61	-	0
TT vs. TC/CC		1	0.98 (0.79, 1.20)	0.81	-	N.A		TT vs. TC/CC	2	0.75 (0.60, 0.93)	0.009	0.06	30
T vs. C		1	0.98 (0.83, 1.17)	0.84	-	N.A		T vs. C	2	0.66 (0.44, 0.98)	0.04	0.28	79
		**IL-17A rs4711998 and CAD**													
**Genetic Model**		**N**	**OR (95% CI)**	**P**	***p*_adj_**	***I*^2^ (%)**							
AA vs. GG		1	0.89 (0.59, 1.33)	0.56	-	N.A							
AA vs. GA		1	0.89 (0.74, 1.08)	0.24	-	N.A							
AA vs. GA/GG		1	0.89 (0.74, 1.07)	0.22	-	N.A							
A vs. G		1	0.91 (0.78, 1.06)	0.24	-	N.A							

N.A: not applicable.

**Table 3:** Egger’s and Begg’s test for the publication bias of rs2275913.

Genetic Models	Egger’s Test *p* Value	Begg’s Test *p* Value
GG vs. AA	0.054	0.462
GG vs. GA	0.041	0.221
GG vs. GA/AA	0.041	0.221
G vs. A	0.029	0.221

## Supplementary Materials

The following are available online at www.mdpi.com/1660-4601/13/7/660/s1 Figure S1: Forest plots of odds ratio for the association of IL-17A rs3819024 with risk of CAD, Figure S2: Forest plots of odds ratio for the association of IL-17A rs3819025 with risk of CAD.
