# Supplementary material for: Meta-Analysis for the Association between Polymorphisms in Interleukin-17A and Risk of Coronary Artery Disease
Source: Int J Environ Res Public Health. 2016 Jun 30;13(7):660. doi: 10.3390/ijerph13070660 (PMC4962201; doi:10.3390/ijerph13070660)
Supplement: Supplementary file 1 [file ijerph-13-00660-s001.pdf]

Supplementary Materials: Meta-Analysis for the Association between Polymorphisms in Interleukin-17A and Risk of Coronary Artery Disease

Mei-Hua Bao, Huai-Qing Luo, Ju Xiang, Liang Tang, Li-Ping Dong, Guang-Yi Li, Jie Zeng and Jian-Ming Li

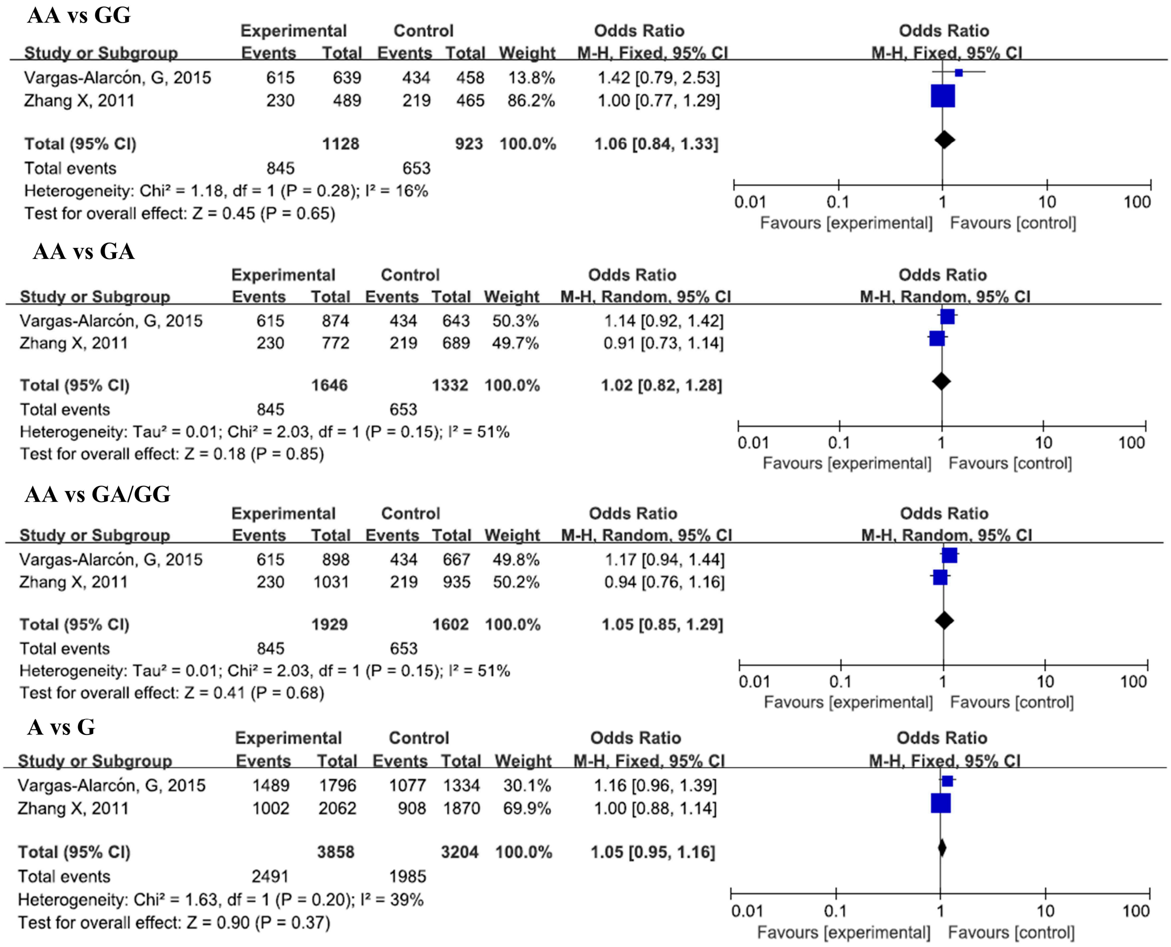

Figure S1. Forest plots of odds ratio for the association of IL-17A rs3819024 with risk of CAD.

AA vs GG

Study or Subgroup	Experimental Events	Experimental Total	Control Events	Control Total	Weight	Odds Ratio M-H, Fixed, 95% CI
Shuang L, 2015	179	238	212	268	59.8%	0.80 [0.53, 1.22]
Zhang X, 2011	635	673	554	593	40.2%	1.18 [0.74, 1.87]
**Total (95% CI)**		**911**		**861**	**100.0%**	**0.95 [0.70, 1.30]**
Total events	814		766			

Heterogeneity: Chi² = 1.47, df = 1 (P = 0.23); I² = 32%
Test for overall effect: Z = 0.31 (P = 0.76)

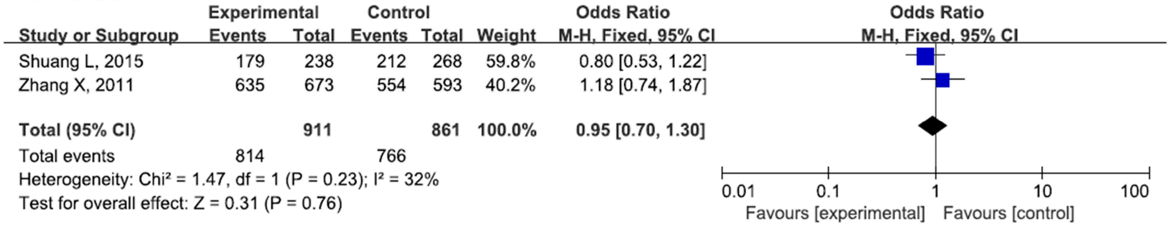

AA vs GA

Study or Subgroup	Experimental Events	Experimental Total	Control Events	Control Total	Weight	Odds Ratio M-H, Fixed, 95% CI
Shuang L, 2015	179	356	212	392	32.3%	0.86 [0.64, 1.14]
Zhang X, 2011	635	993	554	896	67.7%	1.09 [0.91, 1.32]
**Total (95% CI)**		**1349**		**1288**	**100.0%**	**1.02 [0.87, 1.19]**
Total events	814		766			

Heterogeneity: Chi² = 1.93, df = 1 (P = 0.16); I² = 48%
Test for overall effect: Z = 0.23 (P = 0.82)

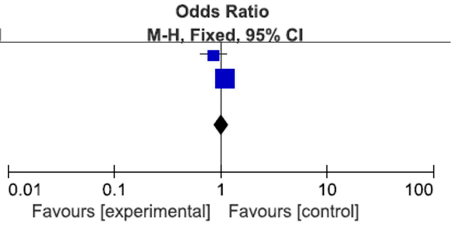

AA vs GA/GG

Study or Subgroup	Experimental Events	Experimental Total	Control Events	Control Total	Weight	Odds Ratio M-H, Random, 95% CI
Shuang L, 2015	635	1031	554	935	57.2%	1.10 [0.92, 1.32]
Zhang X, 2011	179	415	212	448	42.8%	0.84 [0.65, 1.10]
**Total (95% CI)**		**1446**		**1383**	**100.0%**	**0.98 [0.76, 1.27]**
Total events	814		766			

Heterogeneity: Tau² = 0.02; Chi² = 2.61, df = 1 (P = 0.11); I² = 62%
Test for overall effect: Z = 0.12 (P = 0.90)

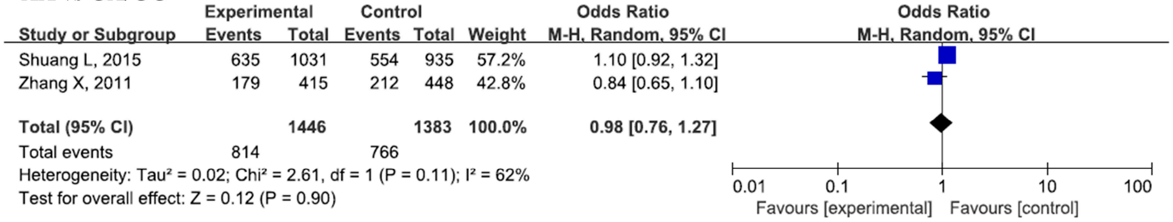

A vs G

Study or Subgroup	Experimental Events	Experimental Total	Control Events	Control Total	Weight	Odds Ratio M-H, Random, 95% CI
Shuang L, 2015	535	830	604	896	45.3%	0.88 [0.72, 1.07]
Zhang X, 2011	1628	2062	1450	1870	54.7%	1.09 [0.93, 1.26]
**Total (95% CI)**		**2892**		**2766**	**100.0%**	**0.99 [0.80, 1.22]**
Total events	2163		2054			

Heterogeneity: Tau² = 0.01; Chi² = 2.82, df = 1 (P = 0.09); I² = 65%
Test for overall effect: Z = 0.13 (P = 0.89)

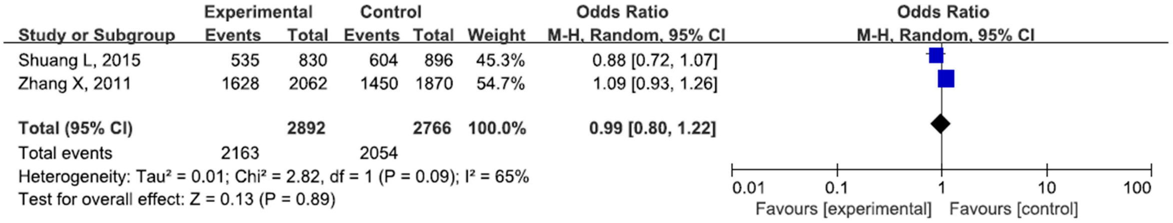

Figure S2. Forest plots of odds ratio for the association of IL-17A rs3819025 with risk of CAD.

© 2016 by the authors; licensee MDPI, Basel, Switzerland. This article is an open access article distributed under the terms and conditions of the Creative Commons by Attribution (CC-BY) license (http://creativecommons.org/licenses/by/4.0/).
